# Multi-omics Mendelian randomisation reveals distinct molecular drivers of pathological myopia complications *vs* simple myopia

**DOI:** 10.7189/jogh.16.04259

**Published:** 2026-10-02

**Authors:** Tong Wang, Aoxiang Wang, Zhongping Chen, Xiaofeng Lin

**Affiliations:** 1 Changsha Aier Eye Hospital, Changsha, Hunan, China; 2 Department of Ophthalmology, The Affiliated Children's Hospital of Xiangya School of Medicine, Central South University, Hunan Children’s Hospital, Changsha, Hunan, China

**Keywords:** myopia, pathological myopia, Mendelian randomisation, proteomics, metabolomics

## Abstract

**Background:**

Pathological myopia complications are leading causes of irreversible blindness, yet the molecular mechanisms distinguishing them from simple myopia remain poorly understood. We aimed to compare the molecular profiles associated with pathological myopia complications and simple myopia using a multi-omics Mendelian randomisation (MR) framework.

**Methods:**

Using circulating protein quantitative trait loci data from the INTERVAL study, plasma metabolite genome-wide association study (GWAS) data from the Nightingale Health – UK Biobank initiative and outcome GWAS data for four complication endpoints and myopia from FinnGen R12, together with self-reported myopia from UK Biobank, we performed a two-sample multi-omics MR study. Molecules were classified as complication-specific, myopia-specific, or shared. We performed sensitivity analyses using MR-Egger, weighted median, Steiger directionality, and reverse MR, and assessed classification robustness through threshold-sensitivity and instrument-stratification analyses.

**Results:**

We identified 872 reliable associations (712 protein and 160 metabolite pairs). Complication-specific proteins (n = 387) outnumbered shared ones (n = 55) by seven to one; similarly, 51 metabolites were complication-specific *vs*. 9 shared. Six lipoprotein lipid ratio–myopia associations survived false discovery rate (FDR) correction (FDR<0.05), all exclusive to simple myopia. Complication-specific proteins were enriched in axon guidance (adjusted *P* = 2.0 × 10^−6^), proteoglycan biosynthesis, and cytokine–cytokine receptor interaction pathways. No robust reverse causation was detected. Top complication-specific targets included endothelin-converting enzyme 1 (odds ratio (OR) = 0.796; *P* = 4.87 × 10^−5^) for retinal detachment with break, vascular endothelial growth factor receptor 2 (OR = 1.441; *P* = 4.94 × 10^−4^) for retinoschisis, and secreted frizzled-related protein 2 (OR = 1.347; *P* = 1.41 × 10^−4^).

**Conclusions:**

Our analysis indicates that molecular drivers of pathological myopia complications are largely distinct from those of simple myopia, with complication-specific targets enriched in neuronal guidance, extracellular matrix metabolism, and inflammatory signalling. These findings may inform therapeutic strategies aimed at preventing the sight-threatening consequences of myopia.

Myopia affects approximately two billion people worldwide, with projections indicating this number would approach five billion by 2050 [1]. While optical correction adequately manages the refractive error itself, a subset of myopic individuals progresses to pathological myopia, a condition characterised by excessive axial elongation and degenerative changes in the posterior segment of the eye [2,3]. The complications of pathological myopia, including chorioretinal atrophy, retinal detachment, myopic macular neovascularisation, and retinoschisis, collectively represent the leading cause of irreversible visual impairment and legal blindness in working-age adults, particularly in East Asia [4,5].

A fundamental question in myopia research is whether the molecular mechanisms driving the initial development of simple myopia are the same as those driving its progression to sight-threatening complications. The prevailing model of myopia pathogenesis centres on a signalling cascade from the retina through the retinal pigment epithelium and choroid to the sclera, culminating in extracellular matrix remodelling and axial elongation [6,7]. However, not all eyes with high myopia – conventionally defined by a refractive threshold (≤−6 dioptres) or axial length (≥26 mm) – develop structural degeneration, so the transition from high myopia to pathological myopia thus likely involves additional molecular insults (*e.g.* vascular insufficiency, accelerated matrix degradation, or inflammatory processes) that are not captured by studies focused on myopia as a binary trait [8,9].

Recent proteome-wide Mendelian randomisation (MR) studies have identified circulating proteins causally associated with myopia [10,11], and metabolomic MR has linked lipid metabolites to refractive error [12]. However, these studies uniformly used simple myopia as their outcome, leaving pathological myopia complications entirely unexplored. This gap is clinically significant because the drug targets most urgently needed are those relevant to complication prevention, rather than myopia onset *per se*. Specifically, atropine and defocus lenses already slow myopia progression, but no pharmacological intervention exists to prevent the degenerative complications that cause blindness [13].

In this study, we performed a multi-omics MR analysis integrating proteomic and metabolomic data to systematically compare the causal molecular profiles of pathological myopia complications with those of simple myopia. By testing 3283 circulating proteins and 249 plasma metabolites against four complication outcomes and two simple myopia outcomes, we aimed to identify complication-specific molecules that could serve as therapeutic targets for preventing the most devastating consequences of myopia.

## METHODS

We conducted a two-sample MR study and report our findings according to the STROBE-MR guidelines [14]. We employed a comparative design in which we tested the same set of molecular exposures (circulating proteins and metabolites) against two groups of outcomes – pathological myopia complications and simple myopia controls – enabling direct comparison of effect profiles across outcome categories. As a secondary analysis of data, we confirm our study aligns with the Journal of Global Health’s GRABDROP guidelines (Table S15 in the **Online Supplementary Document**)

### Exposures, outcomes, and instrumental variables

We obtained the genetic instruments for circulating protein levels from the plasma proteome protein quantitative trait loci (pQTL) resource compiled by Sun *et al*. [15]. This resource, which is based on the INTERVAL study and accessed through IEU OpenGWAS, includes 3,283 protein assays. Instrument selection was not restricted to cis-acting variants. We drew the genetic instruments for plasma metabolites from the Nightingale Health metabolomics genome-wide association study (GWAS) [16], which provides results for approximately 249 metabolites and metabolite ratios measured by nuclear magnetic resonance spectroscopy in over 100,000 participants from the UK Biobank. After instrument selection and harmonisation, 249 metabolites and metabolite ratios were included in the MR analyses across six outcomes. The Nightingale platform predominantly measures lipid and lipoprotein species [16]; we thus performed pathway enrichment only for the proteomic layer.

We obtained outcome summary statistics from six myopia-related GWAS datasets (**Table 1**). Complication outcomes (four endpoints) comprised degenerative myopia (FinnGen R12, H7_DEGENMYOPIA), retinal detachment (H7_RETINALDETACH), retinal detachment with retinal break (H7_RETINALDETACHBREAK), and retinoschisis and retinal cysts (H7_RETINOCHISISCYST), defined by ICD-10 codes in the FinnGen consortium [17]. Simple myopia outcomes (two endpoints) comprised FinnGen myopia (H7_MYOPIA) and self-reported myopia from the UK Biobank (ukb-b-6353; approximately 460,000 participants) [18].

**Table 1:** Data sources for the multi-omics MR study

Data layer	Data source	Platform/consortium	Number of exposures/outcomes	Sample size	Population
Proteomics (exposure)	Sun *et al.* [15]	INTERVAL (SomaScan)	3283 proteins	n = 3301	European
Metabolomics (exposure)	Nightingale Health GWAS [16]	NMR spectroscopy	249 metabolites	n ≈ 115,000	European
Degenerative myopia	FinnGen R12 (H7_DEGENMYOPIA)	ICD-10 coded registry		Total n ≈ 400,000	Finnish/European
Retinal detachment	FinnGen R12 (H7_RETINALDETACH)	ICD-10 coded registry		Total n ≈ 400,000	Finnish/European
Retinal detachment with a break	FinnGen R12 (H7_RETINALDETACHBREAK)	ICD-10 coded registry		Total n ≈ 400,000	Finnish/European
Retinoschisis	FinnGen R12 (H7_RETINOCHISISCYST)	ICD-10 coded registry		Total n ≈ 400,000	Finnish/European
Myopia (FinnGen)	FinnGen R12 (H7_MYOPIA)	ICD-10 coded registry		Total n ≈ 400,000	Finnish/European
Myopia (UK Biobank)	UK Biobank (ukb-b-6353) [18]	Self-reported questionnaire		n ≈ 460,000	European

GWAS – genome-wide association study, MR – Mendelian randomisation, NMR – nuclear magnetic resonance, pQTL – protein quantitative trait loci

For each protein or metabolite, we selected single nucleotide polymorphisms (SNPs) as genetic instruments based on genome-wide significant association with the exposure (*P* < 5 × 10^−8^), low linkage disequilibrium (*r*^2^ < 0.001, 10 Mb clumping window), and sufficient instrument strength (F-statistic >10).

### Statistical analysis

We used the inverse variance weighted (IVW) method as the primary estimator for exposures with two or more SNP instruments [19]. For single-instrument exposures, we applied the Wald ratio. We performed sensitivity analyses for associations with three or more instruments and included MR-Egger regression with intercept test for directional pleiotropy [20], the weighted median estimator [21], and the weighted mode estimator. We assessed heterogeneity using Cochran’s Q statistic and verified causal direction using the Steiger directionality test [22]. We addressed multiple testing by applying the Benjamini–Hochberg false discovery rate (FDR) correction within each omics layer. We additionally applied global Benjamini–Hochberg FDR and Bonferroni corrections across all 13,946 exposure–outcome tests.

To exclude reverse causation, we performed reverse MR for all 55 shared proteins (*i.e.* those associated with both complication and simple myopia outcomes). Myopia GWAS-significant variants (*P* < 5 × 10^−8^) from the UK Biobank were used as instruments, with circulating protein levels as outcomes.

#### Complication-specific *vs*. shared classification

For each omics layer, we classified molecules reaching nominal significance (*P* < 0.05) in at least one complication outcome as complication-associated, with those significant in at least one simple myopia outcome being considered myopia-associated. We designated molecules appearing in both groups as shared; those in only one group were classified as complication-specific or myopia-specific. To assess classification robustness, we repeated this procedure at *P*-values thresholds of <0.01 and <0.001 and in the multi-instrument-only subset (≥2 SNPs).

#### Pathway enrichment analysis

We mapped protein names to gene symbols using the UniProt REST API (458 of 558 unique proteins successfully mapped). We performed gene ontology (GO) biological process and Kyoto Encyclopedia of Genes and Genomes (KEGG) pathway enrichment analyses for complication-specific and myopia-specific gene sets using the ‘clusterProfiler’ *R* package [23] with the Benjamini–Hochberg FDR correction. We conducted comparative enrichment analysis using the compareCluster function. Pathway enrichment was performed only for the proteomic layer, as metabolites cannot be directly mapped to gene ontology terms, and was repeated as a sensitivity analysis using only multi-instrument complication-specific proteins.

#### Drug target identification, repurposing analysis, and colocalisation analysis

To identify potential therapeutic agents, we cross-referenced the prioritised complication-specific proteins against the DrugBank, version 5.1.10 [24] and the Therapeutic Target Database, 2024 update [25]. We identified FDA-approved drugs or those in clinical trials that act as inhibitors or modulators of these target proteins

We performed colocalisation analyses for four prioritised proteins: endothelin-converting enzyme 1 (ECE1), kinase insert domain receptor/vascular endothelial growth factor receptor 2 (KDR/VEGFR2), secreted frizzled-related protein 2 (SFRP2), and ring finger protein 8 (RNF8). Regional plasma pQTL summary statistics were obtained from Sun and colleagues’ SomaScan study [15], and outcome summary statistics for the corresponding endpoints were obtained from FinnGen R12 [17]. For each protein-outcome pair, we applied the ‘coloc’ *R* package [26] to variants within a ±500 kb window centred on the lead pQTL, using the default prior probabilities.

#### Software

We performed all MR analyses in *R*, version 4.5 (R Foundation for Statistical Computing, Vienna, Austria) using the ‘TwoSampleMR’ package [27]. The pathway analyses utilised ‘clusterProfiler’ *R *package with ‘org.Hs.eg.db’. We accessed FinnGen R12 summary statistics locally, and UK Biobank outcomes through the IEU OpenGWAS application programming interface.

## RESULTS

We identified 872 reliable molecule–outcome associations at nominal significance (*P* < 0.05) across two omics layers and six outcomes, comprising 712 associations involving 577 unique circulating proteins and 160 associations involving 126 unique metabolites (**Figure 1**, Panel A; Figure S1 and Table S1 in the **Online Supplementary Document**). The complication outcomes collectively yielded 580 associations, compared with 292 for simple myopia outcomes. Among the 872 associations, 436 (50.0%) were instrumented by a single SNP (Wald ratio) and 436 (50.0%) by two or more SNPs (Table S2 in the **Online Supplementary Document**). MR-Egger intercept *P*-values were available for 254 of the 268 evaluated pairs; 249 (98.0%) showed no evidence of directional pleiotropy (*P*-value for intercept > 0.05; Table S3 in the **Online Supplementary Document**). The Steiger directionality test confirmed the correct causal direction for all tested pairs (Table S4 in the **Online Supplementary Document**). When we applied global multiple testing correction across all 13,946 exposure–outcome tests, no individual association reached significance after either global Benjamini–Hochberg FDR correction (minimum *q* = 0.095) or Bonferroni correction (minimum adjusted *P* = 0.1189) (Table S13 in the **Online Supplementary Document**).

**Figure 1:**
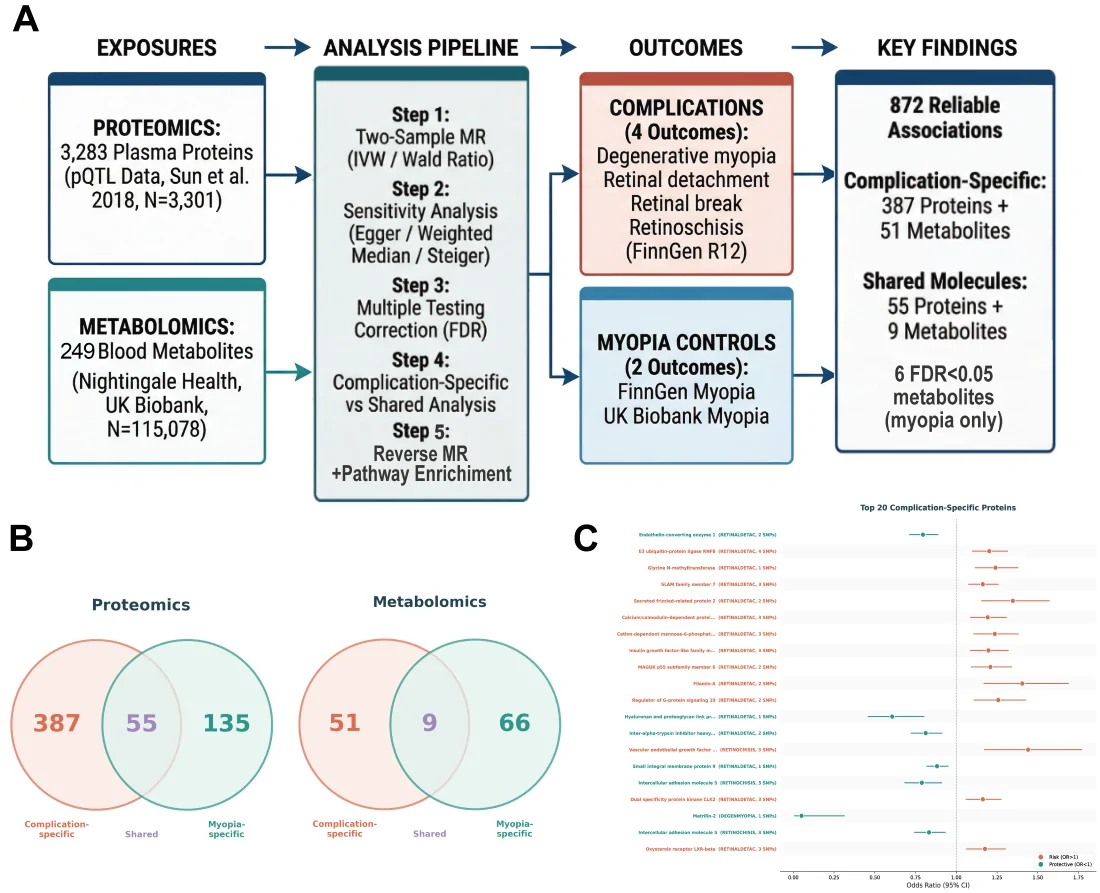
Study overview and complication-specific *vs*. shared molecular profiles. **Panel A.** Study design and analytical workflow. We tested genetic instruments for 3283 circulating proteins and 249 plasma metabolites against four pathological myopia complication outcomes and two simple myopia outcomes using two-sample MR. **Panel B.** Venn diagrams showing the overlap between molecules associated with complications and simple myopia in the proteomic layer (left: 387 complication-specific, 55 shared, 135 myopia-specific) and metabolomic layer (right: 51 complication-specific, 9 shared, 66 myopia-specific). **Panel C.** Forest plot of the top 20 complication-specific proteins, ranked by *P*-value, showing odds ratios and 95% confidence intervals. Red indicates risk factors (OR > 1), teal indicates protective factors (OR < 1). CI – confidence interval, MR – Mendelian randomisation, OR – odds ratio.

### Complication-specific molecules outnumber shared ones by seven to one

In the proteomic layer, 387 proteins were complication-specific (significant only in ≥1 complication outcome), 135 were myopia-specific, and only 55 were shared between both groups. The metabolomic layer showed a parallel pattern: 51 metabolites were complication-specific, 66 myopia-specific, and 9 shared (**Figure 1**, Panel B; Table S5 in the **Online Supplementary Document**). The preponderance of complication-specific molecules – outnumbering shared ones approximately seven to one in the proteomic layer – indicates that the molecular pathology of myopia complications is substantially distinct from that of myopia onset. This pattern was robust to threshold selection: at *P* < 0.01, the proteomic layer retained 94 complication-specific proteins *vs*. only 4 shared (ratio 23.5:1), while at *P* < 0.001, 15 complication-specific proteins remained with zero shared (Table S11 in the **Online Supplementary Document**). The pattern persisted in the multi-instrument protein-only subset, with 162 complication-specific and 19 shared proteins (Table S12 in the **Online Supplementary Document**).

### Six metabolite–myopia associations remained significant after the FDR correction

After FDR correction, six associations reached significance (FDR-adjusted *P* < 0.05), all involving lipoprotein lipid ratios and the UK Biobank myopia outcome (Table S1 in the **Online Supplementary Document**). The ratios of free cholesterol to total lipids in medium and very small very low-density lipoprotein particles showed the strongest evidence (FDR-adjusted *P* = 0.015 for each), followed by triglyceride to total lipid ratios in multiple lipoprotein subclasses. No association with pathological myopia complications were significant after the FDR correction. Beyond these FDR-significant simple-myopia signals, the broader set of nominally complication-specific metabolites, comprising 51 metabolites across 66 associations (Table S10 in the **Online Supplementary Document**), was dominated by lipoprotein subclass traits – high-density lipoprotein-related (n = 19), very low-density lipoprotein-related (n = 16) and low-density lipoprotein-related (n = 7) lipid and lipid-ratio measures – together with fatty acids (n = 9) and a smaller group of non-lipoprotein Nightingale metabolites (n = 11). Many of these associations were inverse (47 of 66) and were distributed across retinal detachment with a break, degenerative myopia, and retinal detachment.

### Top complication-associated proteins

Among proteins associated with complication outcomes, ECE1 showed the strongest evidence for a protective effect on retinal detachment with a break (two instruments: odds ratio (OR) = 0.80; 95% confidence interval (CI) = 0.71–0.89, *P* = 4.87 × 10^−5^). E3 ubiquitin-protein ligase RNF8 was associated with increased risk of retinal detachment with a break (four instruments: OR = 1.20; 95% CI = 1.10–1.31, *P* = 4.89 × 10^−5^). Furthermore, KDR/VEGFR2 emerged as a risk factor specifically for retinoschisis (three instruments: OR = 1.44; 95% CI = 1.17–1.77, *P* = 4.94 × 10^−4^). When examining degenerative myopia specifically, matrilin-2 demonstrated a strong protective effect (one instrument: OR = 0.05; 95% CI = 0.01–0.31, *P* = 1.35 × 10^−3^). Additional prioritised complication-specific proteins included SFRP2 (OR = 1.35; 95% CI = 1.16–1.57; *P* = 1.41 × 10^−4^), which was related to a risk for retinal detachment with a break, and hyaluronan and proteoglycan link protein 1 (OR = 0.61; 95% CI = 0.46–0.80; *P* = 4.30 × 10^−4^), which was protective for retinal detachment (**Table 2**). Colocalisation analysis of four prioritised proteins using Sun and colleagues’ SomaScan regional pQTL data [15] showed moderate support for a shared causal variant between ECE1 (prot-a-883) and retinal detachment with a break (posterior probability for hypothesis 4 = 0.543). The KDR cis-pQTL sensitivity analysis also showed moderate evidence (PP.H4 = 0.469); SFRP2 (PP.H4 = 0.144) and RNF8 (PP.H4 = 0.121) showed lower colocalisation support (Table S14 in the **Online Supplementary Document**).

**Table 2:** Top five prioritised complication-specific proteins driving each severe myopia-related outcome, identified by proteome-wide MR

Protein (gene) for different complications	Number of SNPs	OR (95% CI)	*P*-value
**Degenerative myopia**			
MATN2	1	0.05 (0.01–0.31)	0.001
CHST5	1	10.10 (2.38–42.94)	0.002
DEFB112	1	0.40 (0.22–0.73)	0.003
EPHB2	1	1.95 (1.24–3.07)	0.004
SERPINA10	2	0.72 (0.57–0.91)	0.005
**Retinal detachment**			
HAPLN1	1	0.61 (0.46–0.80)	<0.001
SMIM9	1	0.88 (0.82–0.95)	<0.001
WFIKKN2	1	1.06 (1.02–1.10)	0.004
ITIH5	2	0.91 (0.85–0.97)	0.005
NDUFS4	1	0.96 (0.94–0.99)	0.005
**Retinal detachment with a break**			
ECE1	2	0.80 (0.71–0.89)	<0.001
RNF8	4	1.20 (1.10–1.31)	<0.001
GNMT	1	1.24 (1.12–1.38)	<0.001
SLAMF7	3	1.16 (1.08–1.26)	<0.001
SFRP2	2	1.35 (1.16–1.57)	<0.001
**Retinoschisis**			
KDR	3	1.44 (1.17–1.77)	<0.001
ICAM5	3	0.79 (0.68–0.91)	<0.001
**B4GAT1**	2	1.23 (1.06–1.43)	0.007
SIGLEC12	1	1.16 (1.04–1.29)	0.006
C1orf210	1	2.70 (1.32–5.51)	0.006

CI – confidence interval, MR – Mendelian randomisation, OR – odds ratio, SNP – single nucleotide polymorphism

### Reverse MR confirms causal direction

We observed no significant reverse causal effects for the 55 proteins shared between simple myopia and complications after Benjamini–Hochberg FDR correction (Table S6 in the **Online Supplementary Document**). The Steiger directionality tests performed on all prioritised associations consistently indicated that the variance explained in the exposure exceeded that in the outcome, supporting the hypothesised causal direction (Table S4 in the **Online Supplementary Document**).

### Pathway enrichment reveals distinct biological themes

The GO enrichment of complication-specific proteins spanning 280 mapped genes (**Figure 2**, Panel A; Table S7 in the **Online Supplementary Document**) highlighted axon guidance (20 genes: adjusted *P* = 1.99 × 10^−6^), neuron projection guidance (20 genes: adjusted *P* = 1.99 × 10^−6^), axonogenesis (25 genes: adjusted *P* = 8.27 × 10^−5^), and proteoglycan biosynthetic process (9 genes: adjusted *P* = 2.50 × 10^−3^). Myopia-specific proteins (86 mapped genes) were enriched in defence response to bacterium. The KEGG analysis identified three significantly enriched pathways among complication-specific proteins: cytokine–cytokine receptor interaction, axon guidance, and cell adhesion molecule interaction (**Figure 2**, Panel B, **Table 3**). No KEGG pathways were significant for myopia-specific proteins (Table S8 in the **Online Supplementary Document**). The GO cellular component analysis of the 55 shared proteins revealed enrichment in extracellular matrix organisation and collagen-containing structures (**Figure 2**, Panel C). In a sensitivity analysis restricted to multi-instrument complication-specific proteins (119 mapped genes), KEGG enrichment retained cytokine–cytokine receptor interaction (adjusted *P* = 0.041) and identified fluid shear stress and atherosclerosis (adjusted *P* = 0.019) as the top pathway. GO biological process terms shifted toward vasculogenesis, cell adhesion, and PI3K signalling, consistent with the vascular and inflammatory themes identified in the primary analysis.

**Figure 2:**
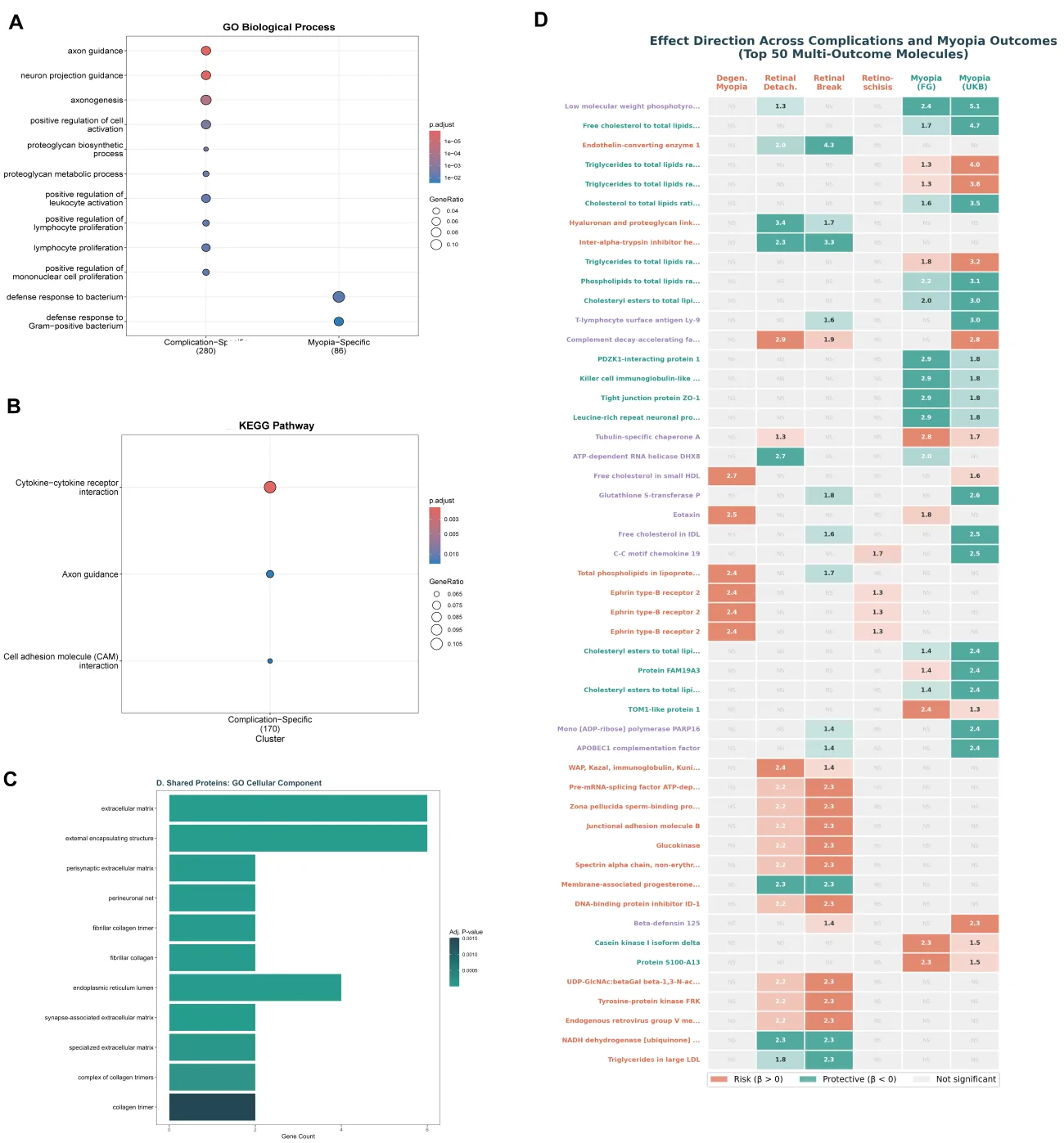
Pathway enrichment and cross-outcome effect patterns. **Panel A.** GO biological process comparison. Complication-specific proteins were enriched in axon guidance, neuron projection guidance, axonogenesis, and proteoglycan biosynthetic process; myopia-specific proteins were enriched in defence response to bacterium. **Panel B.** KEGG pathway comparison. Three pathways were significantly enriched among complication-specific proteins. No significant KEGG pathways were identified for myopia-specific proteins. **Panel C.** GO Cellular Component enrichment of shared proteins, showing enrichment in extracellular matrix and collagen-containing structures. **Panel D.** Heatmap showing the effect direction (red, positive; teal, negative) and −log_10_(P) of the top 50 multi-outcome molecules across six outcomes. GO – gene ontology, KEGG – Kyoto Encyclopedia of Genes and Genomes.

**Table 3:** KEGG pathway enrichment of complication-specific proteins

KEGG pathway	Gene count	Gene ratio	*P* value	Adjusted *P* value	Representative genes
Cytokine–cytokine receptor interaction	18	18/170	7.19 × 10^−6^	1.98 × 10^−4^	IL3, IL15RA, IL31, IL36A, IL1B, CCL1, IFNGR1, SLAMF7, etc.
Axon guidance	12	12/170	1.25 × 10^−4^	1.39 × 10^−2^	EPHB2, EPHB3, EPHA4, SEMA3A, SEMA6A, SEMA7A, UNC5C, NTN4, etc.
Cell adhesion molecule (CAM) interaction	11	11/170	1.51 × 10^−4^	1.39 × 10^−2^	ICAM2, ICAM5, PECAM1, JAM2, CEACAM1, ESAM, etc.

CAM – cell adhesion molecule, KEGG – Kyoto Encyclopedia of Genes and Genomes

### Cross-outcome heatmap reveals distinct effect patterns

Visualisation of the effect direction across all six outcomes for multi-outcome molecules revealed two patterns (**Figure 2**, Panel D). A subset of molecules showed consistent effects across both complication and myopia outcomes, while a larger set showed significance confined to complications or to myopia, reinforcing the complication-specific signature.

### Drug repurposing and therapeutic target identification

As an exploratory drug-target annotation, we identified direct or near-direct matches for a subset of MR-prioritised proteins (Table S9 in the **Online Supplementary Document**). These included elotuzumab targeting SLAMF7, adalimumab and infliximab targeting TNF, ramucirumab and sorafenib targeting KDR/VEGFR2, canakinumab targeting IL-1β, and denosumab targeting TNFSF11/RANKL. Denosumab annotated a shared target between simple myopia and degenerative myopia, whereas the other matches involved complication-specific proteins.

## DISCUSSION

This is the first systematic comparison of the causal molecular profiles of pathological myopia complications and simple myopia that integrates proteomic and metabolomic evidence through MR. Our principal finding is that complication-specific proteins outnumber shared ones by seven to one, and the enriched biological processes differ markedly between the two groups, suggesting that preventing myopia complications may require different therapeutic strategies from those used to prevent myopia itself. Based on these findings, we propose a unified molecular model illustrating the transition from simple axial elongation to advanced pathological remodelling (**Figure 3**). Although no individual association survived global FDR or Bonferroni correction across all tests, the consistency of the complication-specific pattern across stricter significance thresholds – increasing from a 7:1 ratio at *P* < 0.05 to 23.5:1 at *P*-value of <0.01 and complete separation at a *P*-value of <0.001 – and the retention of pathway-level enrichment in the multi-instrument subset together support the biological plausibility of the complication-specific signature.

**Figure 3:**
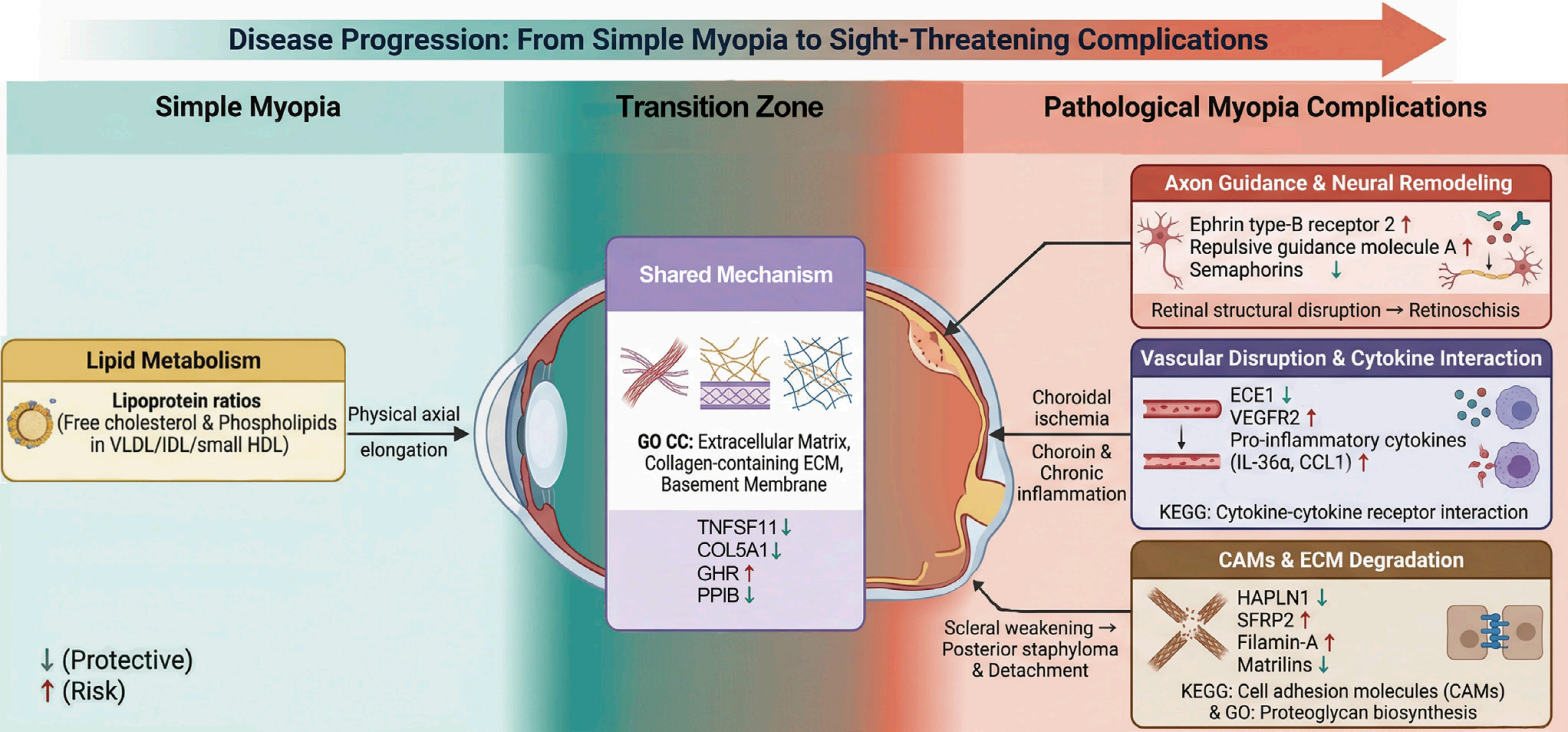
Proposed molecular model of disease progression from simple myopia to pathological complications. Mechanism diagram illustrating the distinct molecular drivers of simple myopia (left: lipid metabolism) *vs*. pathological complications (right: extracellular matrix degradation, vascular disruption, immune/inflammation, scleral remodelling), with shared mechanisms (extracellular matrix organisation) bridging both disease stages.

The 55 shared proteins, though far fewer in number than complication-specific ones, provide mechanistic insight into the biological foundation common to both myopia and its complications. The GO cellular component analysis showed that these shared proteins converge on extracellular matrix organisation, collagen-containing structures, and basement membrane components. This is consistent with the central role of scleral extracellular matrix remodelling in axial elongation [6] and suggests that a subset of ECM-related molecular changes initiated during myopia development persists and contributes to later complications. Among the shared proteins, TNFSF11 (the target of denosumab) and collagen-related molecules may represent the molecular bridge between early scleral weakening and the subsequent posterior staphyloma formation that predisposes to complications [9]. However, an alternative interpretation is that some shared proteins may reflect downstream responses to axial elongation rather than upstream causal drivers. The reverse MR analysis detected no significant causal effects from myopia to shared protein levels (Table S6 in the **Online Supplementary Document**), favouring an upstream role, though reverse MR has limited power. Notably, the FinnGen complication and myopia cohorts likely share participants, which would tend to inflate the number of shared molecules; the predominance of complication-specific associations despite this overlap further supports the distinctness of the two molecular profiles.

The enrichment of complication-specific proteins in axon guidance and neuron projection guidance is notable. Axon guidance molecules, including semaphorins, ephrins, and netrins, play established roles in retinal ganglion cell development and visual circuit formation [28]. Their enrichment among complication-specific proteins suggests that perturbation of neuronal guidance mechanisms may contribute to retinal structural abnormalities – such as retinoschisis and tractional maculopathy – that characterise pathological myopia. The concurrent enrichment of proteoglycan biosynthesis aligns with the known importance of proteoglycans in maintaining scleral biomechanics and vitreoretinal adhesion [6], both compromised in eyes with posterior staphyloma. The KEGG results reinforced these themes, with cytokine–cytokine receptor interaction among the top pathways, providing genetic evidence for the emerging inflammatory hypothesis of myopic retinopathy [29].

In our analysis, VEGFR2 showed a risk effect for retinoschisis (OR = 1.441; 95% CI = 1.17–1.77), while a previous MR study reported VEGFR2 as protective against simple myopia [10]. This difference in effect direction warrants further investigation; whether it reflects differences in the underlying biology remains uncertain. Colocalisation analysis at the KDR cis-pQTL locus showed moderate support (PP.H4 = 0.469), suggesting that the MR signal may partly reflect a shared causal variant, though replication with larger pQTL data sets is warranted.

Endothelin-converting enzyme 1 emerged as the most significant complication-specific protein, with a protective effect on retinal detachment. The endothelin system regulates choroidal blood flow and scleral fibroblast behaviour [30], and our finding suggests that adequate endothelin-mediated vascular regulation helps maintain posterior segment integrity in highly myopic eyes. This target warrants investigation in preclinical models of pathological myopia, such as the LRP2-deficient mouse which develops posterior staphyloma and chorioretinal degeneration resembling human pathological myopia [31,32]. Colocalisation using Sun and colleagues’ SomaScan regional data [15] showed moderate support for ECE1 and retinal detachment with a break (PP.H4 = 0.543), providing additional, though not definitive, evidence for a shared causal mechanism at this locus.

Among the metabolomic findings, the six FDR-significant associations all involved lipoprotein lipid ratios and were exclusive to simple myopia. The metabolomic layer was based on the Nightingale Health platform, which predominantly measures lipid and lipoprotein species; consequently, the 51 complication-specific and 66 myopia-specific metabolites both primarily reflect lipid biology. Nevertheless, the complementary insights from the two omics layers – proteins implicating structural and inflammatory processes, metabolites implicating lipid metabolism – demonstrate the value of the multi-omics approach, and the near-complete separation of complication-specific from myopia-specific metabolites further supports the central finding of distinct molecular drivers (Table S10 in the **Online Supplementary Document**).

Several drug-target annotations emerged from our analysis (Table S9 in the **Online Supplementary Document**). Notably, all matched agents are systemic drugs (*e.g.* oncology and immunology biologics), rather than locally delivered ophthalmic agents; translating any of them to ophthalmic use would therefore require careful consideration of ocular bioavailability and the risk–benefit profile of systemic therapy for a non-life-threatening condition. These annotations should be considered hypothesis-generating and not as evidence for therapeutic intervention.

This study has several strengths. The comparative design is novel in the myopia MR literature and enables insights that single-outcome studies cannot provide. The use of two omics layers offers complementary biological perspectives. Reverse MR confirmed the causal direction for shared proteins, and multiple sensitivity analyses supported the robustness of findings. Several limitations warrant acknowledgement. First, no individual association survived global multiple-testing correction across all 13,946 tests (minimum Benjamini–Hochberg *q* = 0.095), and the nominal classification should therefore be considered hypothesis-generating; moreover, 50.0% of associations relied on a single instrument, precluding sensitivity analyses, and colocalisation analysis showed only moderate support for ECE1 (PP.H4 = 0.543), with no pair reaching the conventional strong threshold (PP.H4 > 0.8). Second, exposure and outcome data derive predominantly from European-ancestry populations, limiting generalisability to East Asian populations where pathological myopia is most prevalent, and circulating protein levels may not faithfully reflect ocular tissue concentrations. Third, the Nightingale metabolomics platform measures predominantly lipid species, and functional validation in experimental models is needed to confirm the identified causal relationships.

## CONCLUSIONS

Our multi-omics MR study indicates that the molecular drivers of pathological myopia complications are largely distinct from those of simple myopia. Complication-specific proteins, enriched in axon guidance, proteoglycan metabolism, and cytokine signalling, outnumber shared molecules by seven to one, while FDR-significant metabolite associations were confined exclusively to simple myopia. These findings suggest that preventing the sight-threatening consequences of myopia may require therapeutic approaches different from those targeting myopia onset, and they identify specific protein targets – including ECE1, VEGFR2, SFRP2, and hyaluronan and proteoglycan link protein 1 – as starting points for complication-focused drug development.

## Additional material


Online Supplementary Document

## Data Availability

**Data availability:** All data used in this study are publicly available. Protein pQTL data were obtained through the IEU OpenGWAS platform (https://gwas.mrcieu.ac.uk/). Metabolomic GWAS summary statistics for Nightingale Health biomarkers were obtained through the IEU OpenGWAS platform (met-d datasets; https://opengwas.io/datasets/).Outcome GWAS data were retrieved from FinnGen R12 (https://www.finngen.fi/) and the UK Biobank(https://www.ukbiobank.ac.uk/). Analytical code is available from the corresponding author upon reasonable request.
