# Supplementary material for: Multi-omics Mendelian randomisation reveals distinct molecular drivers of pathological myopia complications vs simple myopia
Source: J Glob Health. 2026 Oct 2;16:04259. doi: 10.7189/jogh.16.04259 (PMC13631931; doi:10.7189/jogh.16.04259)
Supplement: Supplementary file 1 [file jogh-16-04259-s001.pdf]

Supplement to: Wang T, Wang A, Chen Z, Lin X. Multi-omics Mendelian randomisation reveals distinct molecular drivers of pathological myopia complications versus simple myopia. J Glob Health. 2026;16:04259.

Figure S1. Volcano plots of molecule–outcome associations.

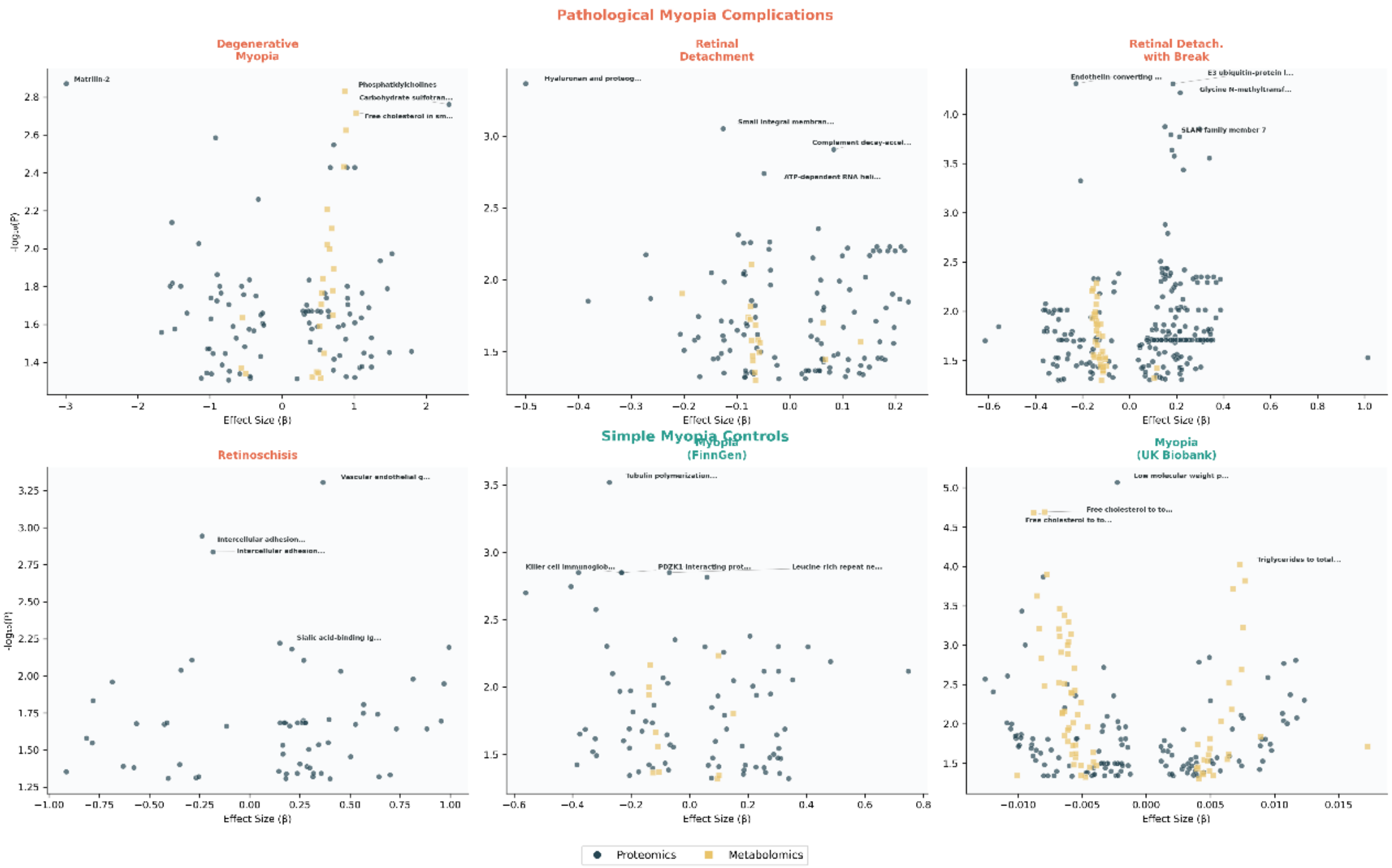

This figure displays the effect size (log OR) against statistical significance (-log10 P) for all six outcomes. Proteomics (circles) and metabolomics (triangles) are distinguished by shape.

Figure S2. Comparison of effect sizes for shared proteins.

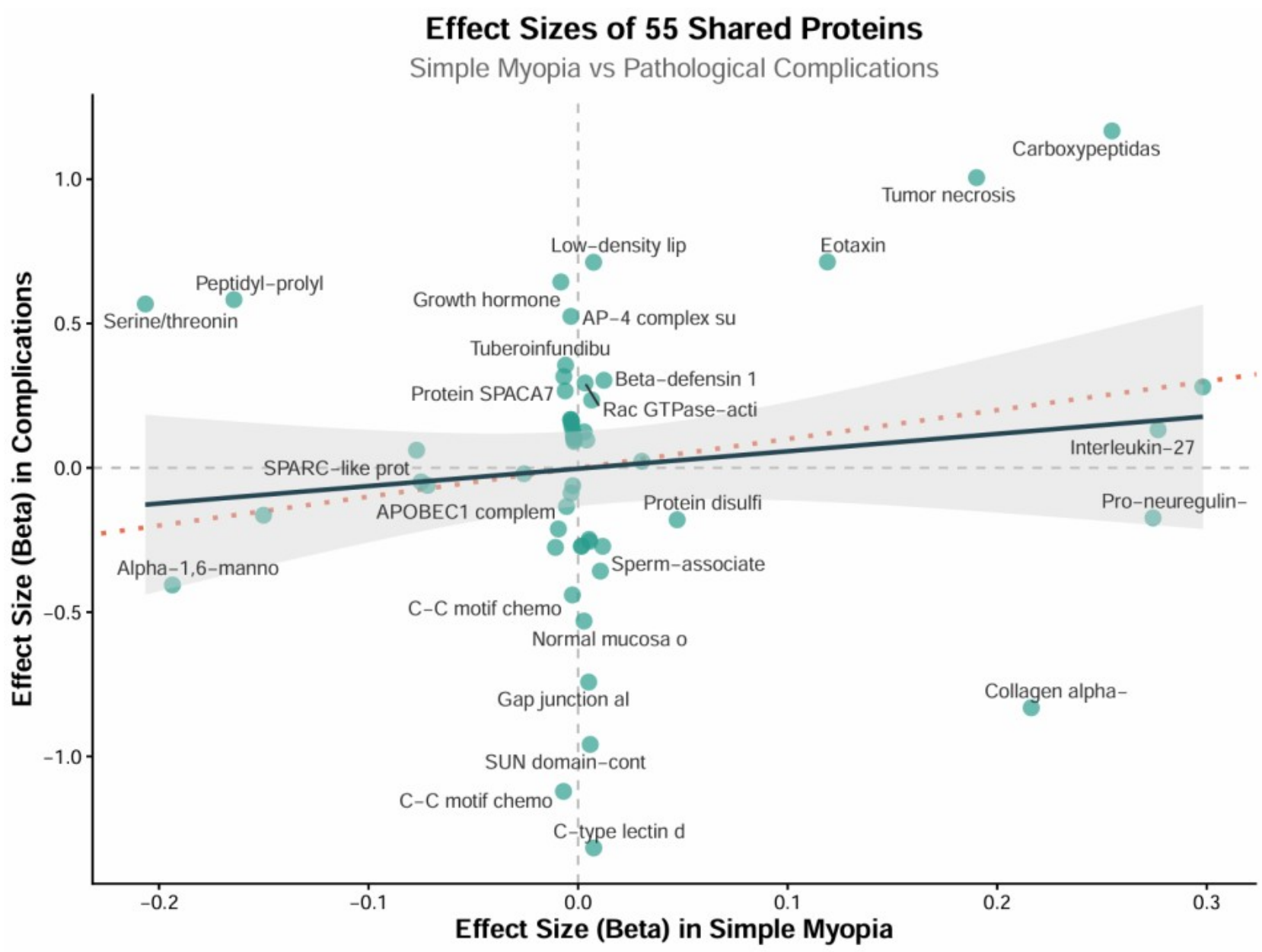

Scatter plot comparing the effect sizes (Beta) of 55 shared proteins in simple myopia (x-axis) versus myopic complications (y-axis).

Table S1. Complete list of 872 reliable molecule–outcome associations.

Note: Due to the extensive size of this dataset, the full table containing comprehensive MR statistics (Beta, SE, P, FDR, etc.) is provided as a standalone digital file (**Table_S1_Full_Results.csv**) in our OSF repository: [Insert OSF Link Here].

Table S2. Multi-method sensitivity analysis results.

Note: This table includes results from IVW, MR-Egger, Weighted Median, and Weighted Mode methods for all testable associations. The full dataset is provided as a standalone file (**Table_S2_Multi_method_sensitivity.csv)** in our OSF repository.

Table S3. MR-Egger intercept test results for prioritized associations.

Outcome	Exposure	Intercept	SE	P-value
RETINALDETACHBREAK	E3 ubiquitin-protein ligase RNF8	0.0477	0.0362	0.32
RETINALDETACHBREAK	SLAM family member 7	0.0562	0.0427	0.41
RETINOCHISISCYST	Vascular endothelial growth factor receptor 2	-0.0727	0.0671	0.48
RETINOCHISISCYST	Intercellular adhesion molecule 5	0.0461	0.1625	0.82
RETINOCHISISCYST	Intercellular adhesion molecule 5	0.0336	0.1228	0.83

Note: Only associations with ≥2SNPs are testable. A P-value > 0.05 indicates no significant horizontal pleiotropy. The full results for all qualifying pairs are available in the OSF repository.

Table S4. Steiger directionality test results for prioritized associations.

Outcome	Exposure	R2 Exposure	R2 Outcome	Direction Correct	P-value
Degenerative Myopia	Carbohydrate sulfotransferase 5	9.16e-03	2.39e-05	TRUE	1.89e-07
Degenerative Myopia	Matrilin-2	1.18e-02	2.51e-05	TRUE	2.53e-09

Degenerative Myopia	Beta-defensin 112	4.15e-02	2.21e-05	TRUE	7.22e-31
Degenerative Myopia	Ephrin type-B receptor 2	5.52e-02	2.05e-05	TRUE	3.91e-41
Degenerative Myopia	Ephrin type-B receptor 2	3.02e-02	2.05e-05	TRUE	1.24e-22
Degenerative Myopia	Ephrin type-B receptor 2	2.40e-02	2.05e-05	TRUE	4.41e-18
Retinal Detachment	NADH dehydrogenase [ubiquinone] iron-sulfur protein 4, mitochondrial	2.42e-01	1.88e-05	TRUE	4.71e-205
Retinal Detachment	Small integral membrane protein 9	4.43e-02	2.70e-05	TRUE	8.29e-33
Retinal Detachment	WAP, Kazal, immunoglobulin, Kunitz and NTR domain-containing protein 2	1.70e-01	1.53e-05	TRUE	1.51e-136
Retinal Detachment	Inter-alpha-trypsin inhibitor heavy chain H5	5.52e-02	1.99e-05	TRUE	3.49e-41
Retinal Detachment	Endothelin-converting enzyme 1	6.60e-02	1.87e-05	TRUE	1.90e-49
Retinal Detachment	WAP, Kazal, immunoglobulin, Kunitz and NTR domain-containing protein 2	2.04e-01	1.98e-05	TRUE	1.27e-167
Retinal Detachment	Hyaluronan and proteoglycan link protein 1	9.28e-03	3.02e-05	TRUE	1.87e-07
Retinal Detachment with Break	Sialic acid-binding Ig-like lectin 12	1.29e-01	1.33e-05	TRUE	1.45e-100
Retinal Detachment with Break	NADH dehydrogenase [ubiquinone] iron-sulfur protein 4, mitochondrial	2.42e-01	1.92e-05	TRUE	5.01e-205
Retinal Detachment	SLAM family member 7	1.09e-01	4.03e-05	TRUE	1.83e-82

with Break					
Retinal Detachment with Break	Inter-alpha-trypsin inhibitor heavy chain H5	5.52e-02	2.98e-05	TRUE	7.57e-41
Retinal Detachment with Break	Glycine N-methyltransferase	7.13e-02	3.92e-05	TRUE	9.06e-53
Retinal Detachment with Break	E3 ubiquitin-protein ligase RNF8	8.66e-02	4.52e-05	TRUE	1.70e-64
Retinal Detachment with Break	Endothelin-converting enzyme 1	6.60e-02	4.12e-05	TRUE	1.12e-48
Retinal Detachment with Break	Endothelin-converting enzyme 1	3.63e-02	2.15e-05	TRUE	5.29e-27
Retinal Detachment with Break	Secreted frizzled-related protein 2	3.27e-02	3.55e-05	TRUE	4.53e-24
Retinal Detachment with Break	WAP, Kazal, immunoglobulin, Kunitz and NTR domain-containing protein 2	2.04e-01	1.02e-05	TRUE	1.73e-168
Retinal Detachment with Break	Hyaluronan and proteoglycan link protein 1	9.28e-03	1.32e-05	TRUE	1.05e-07
Retinal Schisis	Vascular endothelial growth factor receptor 2	0.1394	0	TRUE	2.72E-108
Retinal Schisis	Intercellular adhesion molecule 5	0.2262	0	TRUE	1.79E-188
Retinal Schisis	Intercellular adhesion molecule 5	0.3137	0	TRUE	3.63E-282
Retinal Schisis	Type III endosome membrane protein TEMP	0.0092	0	TRUE	1.42E-07
Retinal Schisis	Sialic acid-binding Ig-like lectin 12	0.242	0	TRUE	4.32E-205
Retinal Schisis	Ephrin type-B receptor 2	0.0552	0	TRUE	1.32E-41
Retinal Schisis	Ephrin type-B receptor 2	0.0302	0	TRUE	5.60E-23

Retinal Schisis	Ephrin type-B receptor 2	0.024	0	TRUE	2.17E-18

Note: 'TRUE' confirms the causal direction from protein to outcome. Full results are available in the OSF repository.

Table S5. Complete molecule classification and gene mapping.

Note: Detailed molecules classification (complication-specific, myopia-specific, or shared) is provided as a standalone file (Table_S5_Molecule_classification_gene_mapping.csv) in the OSF repository.

Table S6. Reverse MR results for 55 shared proteins.

The full dataset is provided as a standalone file (Table_S6_Reverse_MR_shared_proteins.csv) in our OSF repository.

Table S7 & S8. Full GO biological process enrichment results.

Note: Complete lists of enriched GO terms for complication-specific (159 terms) and myopia-specific protein sets are provided as standalone files (Table_S7_GO_complication_specific_proteins.csv and Table_S8_GO_myopia_specific_proteins.csv) in the OSF repository.

Table S9. Drug target matches for complication-specific proteins.

Target protein	Gene	Associated outcome	Matched agent(s)	Match type
SLAM family member 7	SLAMF7	Retinal Detachment with Break	Elotuzumab	Direct target match
Tumor necrosis factor	TNF	Degenerative Myopia	Adalimumab;	Direct target match

			infliximab	
VEGFR2	KDR	Retinoschisis/ Retinal Cyst	Ramucirumab; sorafenib	Direct target match for ramucirumab; multi-target kinase match for sorafenib
TNFSF11 / RANKL	TNFSF11	Degenerative Myopia; Simple Myopia	Denosumab	Direct target match
Interleukin-1 beta	IL1B	Retinal Detachment with Break	Canakinumab	Direct target match

Table S10. All complication-specific metabolite-outcome associations with metabolite class annotation.

The full table of 66 strictly complication-specific metabolite-outcome associations is provided as a standalone XLSX file（Table_S10_Complication_specific_metabolites.xlsx）in OSF.

Table S11. Threshold-sensitivity classification at P < 0.05, P < 0.01, and P < 0.001.

Threshold	Omics	Classification	No. molecules
P < 0.05	Metab.	Complication-Specific	51
P < 0.05	Metab.	Myopia-Specific	66
P < 0.05	Metab.	Shared	9
P < 0.05	Prot.	Complication-Specific	387
P < 0.05	Prot.	Myopia-Specific	135
P < 0.05	Prot.	Shared	55
P < 0.01	Metab.	Complication-Specific	13
P < 0.01	Metab.	Myopia-Specific	38
P < 0.01	Prot.	Complication-Specific	94
P < 0.01	Prot.	Myopia-Specific	50
P < 0.01	Prot.	Shared	4
P < 0.001	Metab.	Myopia-Specific	17
P < 0.001	Prot.	Complication-Specific	15
P < 0.001	Prot.	Myopia-Specific	5

Table S12. Multi-instrument-only subset classification and pathway enrichment results.

Omics	Classification	No. molecules
Metab.	Complication-Specific	51
Metab.	Myopia-Specific	66
Metab.	Shared	9

Prot.	Complication-Specific	162
Prot.	Myopia-Specific	51
Prot.	Shared	19

The full multi-instrument GO biological process and KEGG enrichment results are provided as additional sheets in the standalone XLSX file and in OSF.

Table S13. Global multiple-testing correction results across all 13,946 tests.

Set	N	Prot.	Metab.	Single	Multi	Egger testable	Min P	Min global BH q
All tests	13946	12452	1494	7282	6664	3393	8.50e-06	0.0954
P<0.05 pre-filter	877	712	165	436	441	257	8.50e-06	0.0954
P<0.01 pre-filter	226	169	57	97	129	84	8.50e-06	0.0954
P<0.001 pre-filter	37	20	17	7	30	23	8.50e-06	0.0954
Omics FDR<0.05	6	0	6	0	6	6	2.02e-05	0.0954
Outcome FDR<0.05	37	4	33	1	36	34	8.50e-06	0.0954
Global FDR<0.05	0	0	0	0	0	0	NA	0.0954
Global Bonf.<0.05	0	0	0	0	0	0	NA	0.0954

Note: 877 represents all nominal P < 0.05 associations; the 872 reported in the main text exclude 5 associations that failed additional quality filters.The all-test correction table containing all 13,946 tests is provided as a second sheet in the standalone XLSX file and in OSF.

Table S14. Colocalization results for prioritised proteins using Sun et al. SomaScan pQTL and FinnGen R12 summary statistics.

Protein	Outcome	Locus	Lead SNP	SNPs	PP.H4	Interpretation
ECE1	RETINALDETACH	lead_pqtl	rs4683701	3157	0.146	no strong colocalization support
ECE1	RETINALDETACHBREAK	lead_pqtl	rs4683701	3157	0.543	moderate colocalization support
ECE1	RETINALDETACHBREAK	lead_pqtl	rs4795433	1709	0.14	no strong colocalization support
KDR	RETINOCHISISCYST	lead_pqtl_trans	rs2519093	4225	0.0198	no strong colocalization support
KDR	RETINOCHISISCYST	cis_sensitivity	rs34231037	4071	0.469	borderline colocalization support

SFRP2	RETINALDETACHBREAK	lead_pqtl	rs967645	1712	0.144	no strong colocalization support
RNF8	RETINALDETACHBREAK	lead_pqtl	rs704	1700	0.121	no strong colocalization support

OSF link: [https://osf.io/zbx9w/overview?view_only=da7c7ef200d941f8b27f99da56d7198d].
